# Gadolinium-Based Nanoparticles for Theranostic MRI-Guided Radiosensitization in Hepatocellular Carcinoma

**DOI:** 10.3389/fbioe.2019.00368

**Published:** 2019-11-27

**Authors:** Pengcheng Hu, Zhequan Fu, Guobing Liu, Hui Tan, Jie Xiao, Hongcheng Shi, Dengfeng Cheng

**Affiliations:** ^1^Department of Nuclear Medicine, Zhongshan Hospital, Fudan University, Shanghai, China; ^2^Shanghai Institute of Medical Imaging, Shanghai, China

**Keywords:** nanoparticles, AGuIX, hepatocellular carcinoma, MRI, theranostic, radiosensitization

## Abstract

**Background:** Radiation therapy (RT) of hepatocellular carcinoma (HCC) is limited by low tolerance of the liver to radiation, whereas radiosensitizers are effective in reducing the required radiation dose. Multimodality gadolinium-based nanoparticles (AGuIX) are small and have enhanced permeability and retention effects; thus, they are very suitable for radiation sensitizer HCC RT. Here, we evaluated the potential value of AGuIX for theranostic MRI-radiosensitization in HCC.

**Methods:** The radiosensitization effects of AGuIX were evaluated via *in vitro* and *in vivo* experiments. Tumor growth, apoptosis imaging, and immunohistochemistry were performed to verify the antitumor effects of RT with AGuIX.

**Results:** *In vitro* evaluation of the efficacy of radiosensitivity of the AGuIX demonstrated that the presence of AGuIX significantly decreased HepG2 cell survival when combined with an X-ray beam. *In vivo* MRI imaging showed the ratio of tumor/liver concentration of the AGuIX was the highest 1 h after intravenous injection. For antitumor effects, we found that the tumor size decreased by RT-only and RT with AGuIX. The antitumor effects were more effective with high-dose AGuIX-mediated RT. Apoptosis imaging and immunohistochemistry both demonstrated that the degree of the cell apoptosis was highest with a high dose of AGuIX-mediated RT.

**Conclusions:** This study provides compelling data that AGuIX can facilitate theranostic MRI-radiosensitization in HCC.

## Introduction

Hepatocellular carcinoma (HCC), the most common liver cancer in the world, only 20–25% can be cured by surgery alone (Torre et al., 2015). Most HCC patients require comprehensive multidisciplinary treatment because they are in advanced or first diagnosed as terminal stage (Lope et al., 2012; Waller et al., 2015). Radiotherapy (RT) based on highly penetrating MeV photons (X-rays and γ-rays) is non-invasive and useful for inoperable tumors. HCC itself is a radiotherapy-sensitive tumor, and thus, radiotherapy plays an important role in comprehensive HCC treatment (Poon, 2011). However, radiotherapy suffers from poor tumor specificity. Photons can damage all tissues, leading to serious side effects on the normal liver tissue surrounding the tumor. These patients often have a background of cirrhosis, making them susceptible to lower doses of radiation than normal liver. The incidence of radiotherapy complications rises with increasing radiation dose in which radiation-induced liver disease (RILD) is a serious threat to patients' lives (Kalogeridi et al., 2015). Therefore, simultaneous enhancing the selectivity of tumor tissues and the bioavailability of radiation are the focus of future cancer radiotherapy.

Radiosensitizers can accumulate in the tumor tissue to increase the sensitivity of tumor cell to radiation, making tumor cells more likely to be killed by lower doses of radiation (Kwatra et al., 2013). Many drugs have been developed as HCC radiosensitizers, and the development of nanoparticles (NPs) is one important step (Kunz-Schughart et al., 2017). The use of NPs with the characteristic of preferential aggregation in tumors (even passively absorbed due to enhanced permeability and retention effects, EPR) can lead to local treatment of solid tumors (Rancoule et al., 2016). Furthermore, it has been proposed that NPs with high Z atoms are promising radiosensitizers because they may exert strong radiosensitizing effects on tumors when they are used in combination with several types of radiation of different energies (Liu et al., 2018). Gold NPs have a radiosensitizing effect on HCC (Zheng et al., 2013; Maniglio et al., 2018). However, radiotherapy in the liver region also damages normal tissues because normal liver tissue also has a high uptake of gold NPs (Balasubramanian et al., 2010). Therefore, to increase the target ratio, PEG-coated gold NPs were designed for HCC treatment. PEG-coated gold NPs increase the histocompatibility of gold NPs and prolong the circulation time *in vivo*. When galactose is coupled to gold NPs, it can recognize the asialoglycoprotein receptor (ASGPR) on the HCC, which improves its ability to bind to HCC and increase radiosensitization (Zhu et al., 2015). However, normal hepatocytes can also express ASGPR, and there is still a risk of RILD. Therefore, new nano-radiosensitizers are needed for HCC RT.

In 2013, Mignot et al. constructed a new type of multifunctional gadolinium nanoparticle, AGuIX, which is small (about 5 nm in diameter) and can be quickly excreted by the kidneys (Mignot et al., 2013). Due to the EPR effect, the liver background of AGuIX is much lower than that of AGuIX in most tumor tissues (Kamaly et al., 2012). With a high number of gadolinium atoms (atomic number 64), these nanoparticles can be used for enhanced magnetic resonance imaging (MRI), as well as a radiosensitization (Sancey et al., 2014).

In addition to the Compton effects and the photoelectric effect, the interaction between gadolinium atoms and X-rays also produces an Auger effect. The excited low-energy Auger electrons locally aggregate. There are more aggregation effects with more gadolinium atoms (Butterworth et al., 2012; Coulter et al., 2013). In addition, these materials have good biosafety and biocompatibility at conventional therapeutic concentrations (Morlieras et al., 2013; Bianchi et al., 2014; Bouziotis et al., 2017). Thus, AGuIX are NP radiosensitizers for integrated diagnosis and treatment of HCC.

Our previous research confirmed AGuIX uptake in the HepG2 cells and defined their biodistribution and pharmacokinetics in HepG2 tumor-bearing nude mice. This also indicated that the AGuIX accumulates in the HepG2 xenografts (Hu et al., 2017). Here, we evaluated the radiosensitization effect of AGuIX on HepG2 cells *in vitro* and performed MRI-guided RT using AGuIX radiosensitizer. We also conducted apoptosis Micro-SPECT/CT imaging to explore the radiosensitizing effect of AGuIX on HepG2 xenograft *in vivo*.

## Materials and Methods

### AGuIX Nanoparticles

Gadolinium nanoparticles (AGuIX) were purchased from Nano-H (Lyon, France). The nanoparticles were spherical, dehydrated and sub-5 nm in diameter. Via built-in DOTA chelators the gadolinium atoms were attached to a polysiloxane shell in AGuIX nanoparticles. Rehydrated in sterile, Diethyl pyrocarbonate (DEPC)-treated water (Invitrogen, USA), AGuIX nanoparticles were stored at 4°C according to the manufacturer's instructions.

### Cell Culture

The human HCC cell line, HepG2, was obtained from the Chinese Type Culture Collection (Chinese Academy of Sciences, Shanghai, China). HepG2 cells were cultured (37°C, 5% CO_2_) in Dulbecco's modified Eagle's medium (Gibco) supplemented with 100 IU/ml penicillin-streptomycin and 10% fetal bovine serum.

### Cell Irradiation With γ-Rays

HepG2 cells incubated in medium were first divided into four groups, and then irradiated at intensity from 1 to 6 Gy. Four different combinations were studied: A. Irradiation without AGuIX. B. AGuIX (0.5 nM) was added in the media just before the irradiation. This combination was called +IR/–incubation. C. Incubated cells with the AGuIX (0.5 nM) for 1 h and the media was changed just before the irradiation. This combination was called +IR/+washing. D. Incubated cells were with the AGuIX (0.5 nM) for 1 h and then irradiated. This combination was called +IR/– washing. The X-ray source (X-RAD 320, Precision X-Ray, North Branford, CT, USA) was used for irradiation. It was operated at 300 kV and 8 mA with a 2-mm Alfilter at a dose rate of 2.0 Gy/min.

### Quantification of AGuIX-Mediated Cell Radiosensitization Effects via a Clonogenic Assay

The cells were washed with PBS, trypsinized, and counted after irradiation. The irradiated cells were incubated at 300 cells per plate and grow for 10 days in 10 cm dishes. They were stained with 10% ethanol dye solution and 1% crystal violet. The clones in plates were counted, and measurements were done in triplicate. Linear-quadratic (LQ) model was used for cell survival curves fitting. The radiation doses reducing cells survival rate to 37% (D1%) on radiation survival curve divided by that on corresponding curves of radiation with AGuIX was called sensitizing enhancement ratio (SER).

### Animal Models

The protocol for animal research was approved by the medical ethics committee of Zhongshan Hospital, Fudan University. This study followed the relevant guidelines and regulations of Fudan University. Six-week-old male BALB/c athymic nude mice weighing between 16 and 18 g were obtained from Slac Biotechnology (Shanghai, China). The mice were subcutaneously injected with HepG2 cells (5 × 10^6^/100 μl) in the right flank.

### *In vivo* MRI

To observe the distribution of nanoparticles *in vivo* and choose appropriate time for radiotherapy, three groups of nude mice-bearing HepG2 tumors with AGuIX (10 mg/200 μl nanoparticles injected into the tail vein) were chosen for MRI imaging. MRI scans were performed at four time points: before injection of nanoparticles, 1 h post injection (p.i.), 3 h p.i., and 6 h p.i. The MRI was a 7 T scanner (BioSpec 70/20 USR, Bruker Biospin MRI GmbH, Germany). We estimated the AGuIX's concentration in the tumor based on the signal of contrast-enhanced T1-weighted images (TR = 650 ms, TE = 700 ms, NA = 1, slice thickness = 0.7 mm, reconstruction voxel size = 256 × 256 × 700 μm, FOV = 3 × 4.25 cm). According to the formula [Gd^3+^∞1/S^T1^(t)−1/S^T1^(t_0_)] where S^T1^(t_0_) is the signal strength before NPs injection and S^T1^(t) is the signal strength at a determined time after NPs injection, the Gd^3+^'s concentration was estimated based on the relaxation determined by the signal strength of contrast-enhanced T1 images (Lux et al., 2011; Detappe et al., 2017).

### Radiotherapy *in vivo*

To verify radiosensitization effect *in vivo*, Twenty four mice bearing subcutaneous right flank tumors were divided into four groups (*n* = 6). The diameter of the tumors were 4–6 mm. The mice in the control group and solely radio therapy group were injected with 0.5 mL of normal saline through the tail vein once a day for 2 days. The mice in the other two group were injected with 1 mg and 10 mg of AGuIX in 0.5 mL of normal saline through the tail vein, respectively. The tumor bearing mice received 6 Gy radiotherapy just 1 h after the intravenous injection on consecutive 2 days. All the mice were irradiated using an X-ray source (X-RAD 320, Precision X-Ray, North Branford, CT, USA) operating at 300 kV and 8 mA with a 2-mm Al filter at a dose rate of 2.0 Gy/min with a 2 × 2 cm radiation field to cover the tumor. Other parts of the body were covered with 5 mm lead shield. Tumor growth was measured over the following days.

### Micro-SPECT/CT Apoptosis Imaging

Twelve HepG2 tumor bearing mice were randomly divided into four groups (*n* = 3) and injected with ^99m^Tc-duramycin (37 MBq/2 μg/mouse) through the tail vein. Two hours after injection, mice were anesthetized using inhalation of 2% isoflurane. Then they were scanned in a simultaneous micro-single-photon emission computed tomography/computed tomography (SPECT/CT) scanner (Bioscan, Washington DC, USA). The SPECT/CT imaging parameters were as follow: SPECT: energy peak, 140 keV; scanning time, 35 s/projection; window width, 10%; resolution, 1 mm/pixel; and matrix, 256 × 256. CT: tube current, 0.15 mA; tube voltage, 45 keV; exposure time, 500 ms/frame, and frame resolution, 256 × 512. The HiSPECT algorithm was used for imaging reconstruction.

InVivoScope software (Version 1.43, Bioscan, Washington DC, USA) was used for imaging post-processing. Two 3D region of interest (ROI) were drawn in the region of the tumor and similar region on the contralateral muscle as background ROI. The concentration of radioactivity of each ROI (μCi/mm^3^) was determined using this software. The tumor-to-background ratio (T/B) was regarded to express tumor signal intensity with reducing inter-mice variations.

### Immunohistochemistry

Mice were sacrificed after apoptosis imaging. Tumors were harvested from the above four groups. The harvested tumors were immersed in 4% paraformaldehyde buffered solution for 24 h. Then dehydrated and embedded in paraffin. The tumor was sectioned serially to 4 mm for TUNEL staining with a TUNEL Apoptosis Assay kit (Roche Diagnostics, Indianapolis, IN, USA) following the manufacturer's protocol. The immunostaining was evaluated by determining the histochemistry score (H-SCORE). H-SCORE = ∑(PI × I) = (percentage of cells of weak intensity × 1) + (percentage of cells of moderate intensity × 2) + (percentage of cells of strong intensity × 3) (Azim et al., 2015; Yeo et al., 2015).

### Statistics

The cell growth curve was analyzed by OriginPro 8.0. Statistical analysis of image data and the tumor diameter used the Student's *t* test for comparisons between two groups by SPSS. In all cases, data were presented as mean ± SD (standard deviation). Statistical significance (^*^) was set at *P* < 0.05.

## Results

### Radiosensitizing Effect Exerted by Irradiated AGuIX *in vitro*

**Figure 1:**
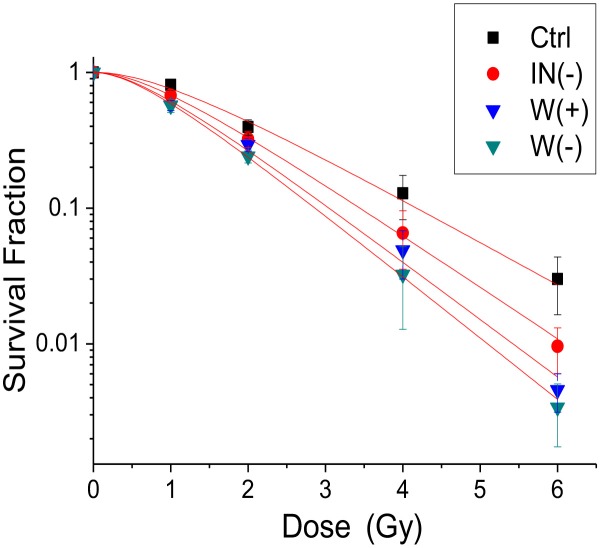
Radiation dose enhancement studies. Clonogenic assays of HepG2 tumor cells post-AGuIX incubation (1 h). Ctrl is the control group with no AGuIX incubation. IN (–) is AGuIX not incubated, W (+) represents AGuIX incubation and washed, W (–) is AGuIX incubation without washing. Linear quadratic models were fitted to experimental data.

Significant radiosensitization effect was observed in all clonogenic assays (Figure 1). The cell cloning rates of the 1-6 Gy experimental groups were lower than that of the control group (*P* < 0.05). This shows that AGuIX has radiosensitization effects on each experimental group. The results of the dose enhancement studies are summarized in Tables 1, 2. The +IR/-washing group showed the strongest radiosensitization followed by the +IR/+washing group, and finally the +IR/-incubation group. The SER values were 1.26, 1.45, and 1.52 for the cells treated with the +IR/- incubation (IN-), the +IR/+washing (W+), and the +IR/- washing (W-), respectively.

**Table 1:** Clonogenic assays.

	**Ctrl**		**IN (–)**		**W (+)**		**W (–)**	
**Dose (Gy)**	**Survival**	**SE**	**Survival**	**SE**	**Survival**	**SE**	**Survival**	**SE**
0	1	1.00E-03	1	0.02	1	0.003	1	0.005
1	0.80907	0.07355	0.67502	0.055	0.57162	0.04764	0.57011	0.06335
2	0.39495	0.05247	0.32314	0.04475	0.29336	0.03154	0.24099	0.02591
4	0.12829	0.04616	0.06544	0.02993	0.04904	0.01908	0.03238	0.0196
6	0.02995	0.01357	0.00957	0.00353	0.00458	0.00144	0.0034	0.00166

*Clonogenic assays of HepG2 tumor cells post-AGuIX incubation (1 h) after RT in Ctrl, IN (–), W (+) and W (–) groups*.

**Table 2:** Dose enhancement effect.

**Ctrl**	**IN (^**−**^)**	**W (+)**	**W (–)**
D0 1.44	1.14	0.99	0.95
SER	1.26	1.45	1.52

*Dose enhancement effects in terms of SER for HepG2 cells incubated with 0.5 mM AGuIX in three groups. The sensitizing enhancement ratio (SER) was determined as the radiation dose reducing the survival to 37% (D0) for the radiation-only survival curve divided by that on corresponding curves of AGuIX plus radiation. SER = D0 (radiation-only group)/D0 (AGuIX plus radiation groups)*.

### MRI Imaging of Tumors *in vivo*

**Figure 2:**
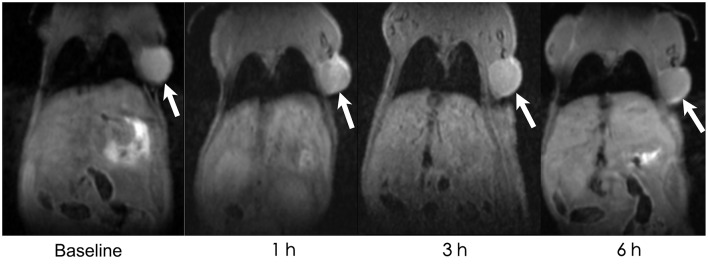
*In vivo* MRI. *In vivo* MRI of the HepG2 tumor (white arrow)-bearing mice before AGuIX injection (baseline), 1, 3, and 6 h after AGuIX injection through the tail vein.

**Figure 3:**
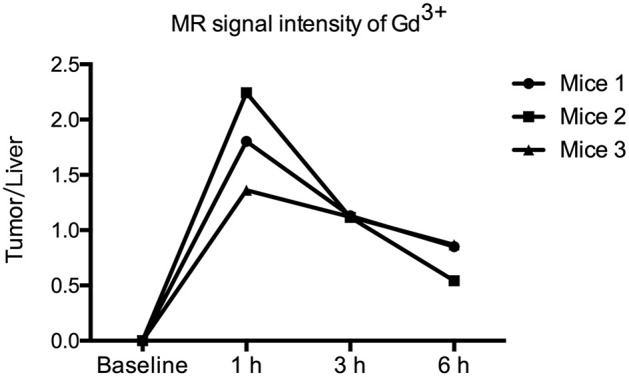
Ratio of the MR signal intensity of Gd^3+^ in the tumor and liver. The T1-weighted MR images were acquired before and 1, 3, and 6 h after intravenous injection of 1 ml of AGuIX NPs ([Gd^3+^] = 100 mM) over three mice (1), (2), and (3). The ratios of the MR signal intensity of Gd^3+^ in the regions of tumor and liver were calculated.

AGuIXs were injected into the mice through the tail vein, and T1-weighted images were scanned at 1, 3, and 6 h (Figure 2). After injection of the AGuIX, we found that the tumor region (white arrow) became brighter than the background tissue and even the liver as seen by coronal scanning (Figure 2). With longer time, both the signal intensity of the tumor and the liver decreased gradually. The concentration of Gd^3+^ in the regions of interest (tumor, liver, muscle) was calculated according to the formula [Gd^3+^∞1/S^T1^(t)-1/S^T1^(t_0_)] (Figure 3). The Gd^3+^ concentration ratio of tumor/liver 1 h after intravenous injection is the highest among 1, 3, and 6 h.

### Radiosensitization Assessment *in vivo*

**Figure 4:**
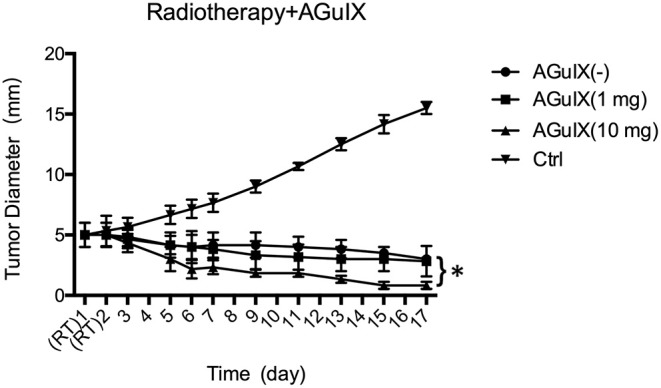
*In vivo* evaluation of the efficacy of radiosensitivity of AGuIX in mice-bearing HepG2 tumors. Tumor growth curves following the different treatment groups including the control group with saline solution (Ctrl), single RT without AGuIX [AGuIX(–)], RT with AguIX (1 mg), (AguIX (1 mg), and RT with AguIX (1 mg) AguIX (10 mg). Each group has 6 mice. The asterisk indicates statistical significance (*p* < 0.05).

The curve of tumor growth showed that the tumors size increased rapidly in the control group during the whole period of investigation and enlarging nearly 3.2-fold in 17 days (Figure 4). In the group treated with radiotherapy alone and with AGuIX (1 mg), the tumor diameter decreased by two with a 6 Gy dose per fraction; however, it showed no statistical difference in tumor diameter throughout the period of investigation. During this process, the tumor diameter in AGuIX (–) group and AGuIX (1 mg) group decreased by ~40 and 44%, respectively. In contrast, AGuIX (10 mg) combined with the radiation group showed that the tumors size shranked by 83%. Versus radiotherapy alone, the introduction of the AGuIX caused a significant dose enhancement effect that inhibited the growth of the tumors.

### Apoptosis Imaging and Immunohistochemistry

**Figure 5:**
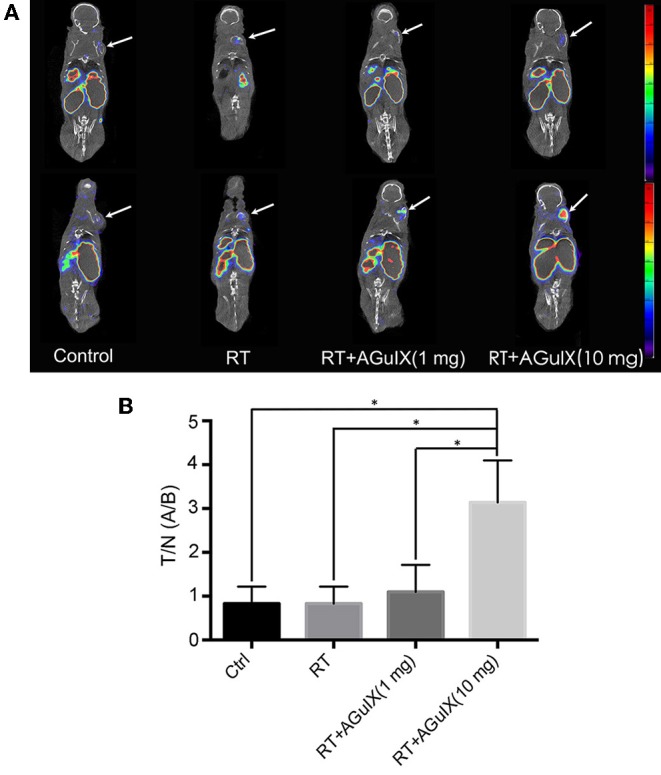
^99m^Tc-duramycin SPECT/CT images of the mice in control group and therapy groups before (upper row) and after (below row) radiation. **(A)** First panel is the control group with no therapy, ^99m^Tc-duramycin SPECT/CT images and 3 days after ^99m^Tc-duramycin SPECT/CT images again. In the rest of the panel, ^99m^Tc-duramycin SPECT/CT images were compared in each panel 1 day before and 1 day after irradiation, and the three panels showed the images of mice injected by tail vein injection of normal saline 1 mg of AguIX (third panel) and 10 mg of AGuIX (fourth panel), respectively (*n* = 3). The same color scale was applied to each of the images. **(B)** The tumor-to-background ratio (T/B) was used to express tumor signal intensity. Independent-sample *t* test was used for statistics. The asterisk indicates statistical significance (*P* < 0.05).

**Figure 6:**
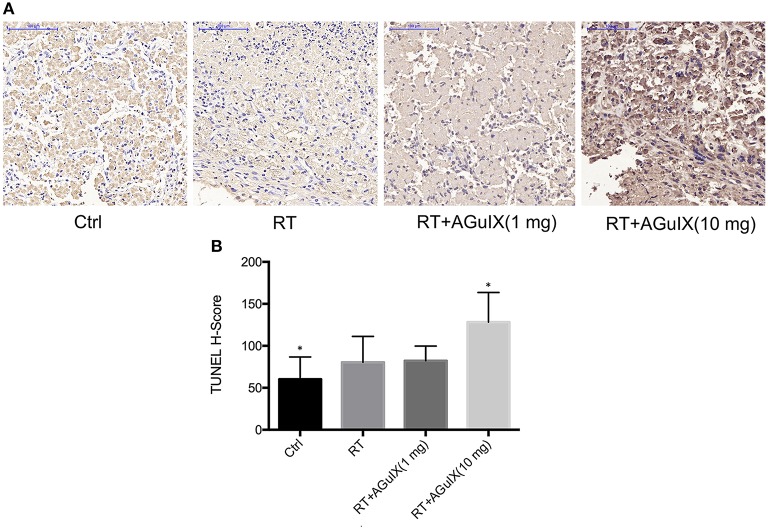
Pathology and immunohistochemistry of tumor tissues. **(A)** Microscopic images of TUNEL (×200) staining of tumor tissues obtained from HepG2-bearing mice with no therapy were used as control. In TUNEL staining, apoptotic cells are stained brown. **(B)** Data analysis using the H-SCORE of TUNEL staining of tumor tissues to compare the four groups. Independent-sample *t* test was used for statistics. The asterisk indicates statistical significance (*P* < 0.05).

The radioactivity of the tumor increased progressively in the group of RT with AGuIX (10 mg) after therapy. The tumor-to-background ratio (T/B) was 0.83 ± 0.39, 1.37 ± 0.31, 1.10 ± 0.62, and 3.87 ± 0.96 in the Control group, RT group, RT + AGuIX (1 mg) group, RT + AGuIX (10 mg) groups, respectively (*n* = 3, mean ± SD). The T/B of the RT + AGuIX (10 mg) was the highest among four groups and showed statistical differences vs. the other three groups (Figure 5). The results of H-SCORE and TUNEL staining of tumor tissues among the 4 groups were consistent with apoptosis imaging; the score of RT + AGuIX (10 mg) was also high. There was a statistical difference between the RT + AGuIX (10 mg) group and the control group (Figure 6).

## Discussions

AGuIX nanoparticles have been proven the capability of increasing the tumor cells' sensitivity to radiation therapy in a number of tumor cells (including radiation-resistant cell lines) *in vitro*. The SERs were observed from 1.1 to 2.5 (Sancey et al., 2014). Besides, AGuIX NPs, the first nanoparticles based on multifunctional silica with a hydrodynamic diameter under 5 nm, are sufficiently small to escape hepatic clearance and allow animal imaging by four complementary techniques (SPECT, fluorescence imaging, MRI and CT) (Lux et al., 2011). Thus, AGuIX has potential to be developed into theranostic MRI-radiosensitization for HCC (Kamaly et al., 2012; Lux et al., 2015).

In order to study the radiosensitization effect of AGuIX on HCC RT, we next irradiated HepG2 cells. Our previous research showed that AGuIX can be taken up into the HepG2 cytoplasm and has a good dispersion shape in HepG2 cells, indicating that AGuIX was stable in the cells (Hu et al., 2017). The obtained of radiation dose enhancement studies in HepG2 cells (Figure 1) are similar to that by Porcel et al. in pancreatic cancer cells (Detappe et al., 2015). Irradiated AGuIX groups presented a stronger inhibition of cell clonogenic rates than the control group.

Radiosensitization was also observed in the groups with AGuIX regardless of whether AGuIX is incubated with cells for 1 h in our study. The mechanism of action of radiotherapy, in addition to the direct killing effect of the incident radiation beam, there may be other factors contributing as well. McMahon et al. demonstrated Auger electrons can create a local effect and affect nanoparticles clustering, which is the main cause of the formation of reaction oxygen species (ROS) such as OH°, H_2_O_2_, or HOCl (McMahon et al., 2011). Also, a biological and chemical effect should be explored to account for the measured radiosensitization as well. Some of these ROS, with high chemical stabilities and a long-range action (few mm), may increase the cell death whether the nanoparticles are in the intercellular space or in cytoplasm. Therefore, the results of the study *in vitro* hinted that AGuIX whether in tumor intercellularly or in tumor intracellularly can both have the radiosensitization effect *in vivo*.

To evaluate the radiosensitization effect of AGuIX *in vivo*, we intravenously injected AGuIX into the HepG2 xenograft mice. For HCC, when the radiosensitizer concentration ratio of tumor/liver was the highest, it is the most suitable time point for RT to maximize therapeutic effect and minimize side effects to normal hepatic tissue. According to this hypothesis, we performed MRI on these mouse models and selected 1 h p.i. mice as the therapeutic time point for RT. The MRI data suggested that the signal intensity and contrast of the tumor lesion was significantly enhanced after 1 h AGuIX post injected. Therefore, MRI could be used for guiding the radiation process by monitoring and assisting tumor localization in real time.

The tumor growth curve in Figure 4 showed that the introduction of the AGuIX during the radiation process result in a markedly dose increasing effect compared to radiotherapy alone, thereby inhibiting the growth of solid tumors. Tumor cell apoptosis after radiotherapy is the basis of cell death (Garcia-Barros et al., 2003; Neshastehriz et al., 2018), and apoptosis imaging *in vivo* can achieve early detection of tumor post-radiation response, as well as accurate prediction of radiotherapy efficacy (Verheij, 2008). The ^99m^Tc-duramycin is a relatively mature apoptosis imaging agent and has been used in apoptotic imaging of various tumors (Johnson et al., 2013; Audi et al., 2015; Elvas et al., 2016). Therefore, ^99m^Tc-duramycin Micro-SPECT/CT imaging was performed to access the antitumor effects of imaging-guided RT with AGuIX. Combined with the apoptosis immunohistochemistry, both results demonstrated that the group of AGuIX-mediated RT showed the highest degree of cell apoptosis. These concurred with the tumor growth data. Thus, apoptosis imaging, pathology and tumor growth data suggest that the EPR-dependent accumulation of AGuIX within tumors can enhance the efficacy of radiation therapy.

## Conclusions

We describe a gadolinium-based nanoparticle AGuIX for MRI-guided radiotherapy in HCC. The AGuIX provide better detection of tumors in imaging, and precise identified for accurate MRI-guided radiotherapy. On the other hand, the heavy elements in this novel nanoparticle can enhance radiosensitizing effect by irradiation dose deposition. The preliminary results showed that the radiosensitizing effect observed *in vivo* could be translated to remarkable tumor control via AGuIX-radiosensitized radiotherapy. Thus, this study demonstrates that the AGuIX could be a promising theranostic nanoparticle for both MRI-guieded and enhancement of radiosensitivity. Thus, it can overcome the limitations of RT in HCC by increasing the tolerance of the liver tumor to radiation.

## Data Availability Statement

All datasets generated for this study are included in the article/supplementary material.

## Ethics Statement

The animal research protocol was approved by Zhongshan Hospital medical ethics committee, Fudan University. All experiments were conducted following the relevant guidelines and regulations of Fudan University.

## Author Contributions

PH, ZF, HT, and HS conceived and designed the study. PH, GL, and JX performed the experiments. PH, HT, JX, and DC were engaged in the data interpretation and analysis. PH, ZF, and DC participated in the writing of the manuscript.

### Conflict of Interest

The authors declare that the research was conducted in the absence of any commercial or financial relationships that could be construed as a potential conflict of interest.
